# Unveiling Molecular Effects of the Secondary Metabolite 2-Dodecanone in the Model Hymenopteran *Nasonia vitripennis*

**DOI:** 10.3390/toxics12020159

**Published:** 2024-02-18

**Authors:** Rosario Planelló, Mónica Aquilino, Laureen Beaugeard, Lola Llorente, Óscar Herrero, David Siaussat, Charlotte Lécureuil

**Affiliations:** 1 Molecular Entomology, Biomarkers and Environmental Stress Group, Faculty of Science, National Distance education University (UNED), 28232 Las Rozas de Madrid, Spain; maquilino@ccia.uned.es (M.A.); lolallorente@ccia.uned.es (L.L.); oscar.herrero@ccia.uned.es (Ó.H.); 2 Institut de Recherche sur la Biologie de l’Insecte (IRBI), CNRS-Université de Tours, 37200 Tours, France; laureen.beaugeard@univ-tours.fr; 3 Institute of Ecology and Environmental Sciences of Paris, Department of Sensory Ecology, Sorbonne Université, Campus Pierre et Marie Curie, 75005 Paris, France; david.siaussat@admp6.jussieu.fr

**Keywords:** methyl ketones, parasitoid wasp, toxicity assays, transcriptional alterations, physiological responses, reproduction

## Abstract

Over the past decade, multiple studies have suggested that the secondary metabolites produced by plants against herbivorous insects could be used as biopesticides. However, as the molecular mechanism of action of these compounds remains unknown, it is difficult to predict how they would affect non-target insects; thus, their innocuity needs to be clarified. Here, we investigate, from the molecular level to the organism, the responses of a useful parasitic insect *Nasonia vitripennis* (Walker, 1836) being exposed at the pupae stage for 48 h (up to 6 days) to sublethal doses (5 µg/L and 500 µg/L) of 2-Dodecanone. 2-Dodecanone altered the gene expression of genes related to ecdysone-related pathways, biotransformation, and cell homeostasis. A significant induction of ecdysone response-genes (*EcR*, *usp*, *E78*, *Hr4*, *Hr38*) was detected, despite no significant differences in ecdysteroid levels. Regarding the cell homeostasis processes, the gene *l(2)efl* was differentially altered in both experimental conditions, and a dose-dependent induction of *hex81* was observed. 2-Dodecanone also triggered an induction of *Cyp6aQ5* activity. Finally, 2-Dodecanone exposure had a significant effect on neither development time, energy reserves, nor egg-laying capacity; no potential genotoxicity was detected. For the first time, this study shows evidence that 2-Dodecanone can modulate gene expression and interfere with the ecdysone signalling pathway in *N. vitripennis.* This could lead to potential endocrine alterations and highlight the suitability of this organism to improve our general understanding of the molecular effects of plant defences in insects. Our findings provide new insights into the toxicity of 2-Dodecanone that could potentially be explored in other species and under field conditions for plant protection and pest management as a means to reduce reliance on synthetic pesticides.

## 1. Introduction

Current agriculture faces the challenge of moving towards more sustainable models where production prioritises human health and the environment. Indeed, pesticides are characterised by varying their toxicity to target and non-target organisms [1,2]. Public opinion is exerting pressure to develop safer agricultural practices, including biopesticides composed of or derived from natural products to replace synthetic molecules [3]. Biorational insecticides are considered less hazardous to the environment because of their expected specificity to their target and their potentially higher degradation rate in the soil compared to synthetic molecules [4,5]. As plant secondary metabolites have evolved as an essential defence against herbivorous insects, the toxicity exerted by some of them could be exploited to be used as biopesticides [6]. To date, xenobiotic plant secondary chemicals that alter insect growth have been observed in many species [7]). For example, many plant species produce volatile essential oils containing one or a combination of methyl ketones (MKs) mixed with other organic compounds to protect the plant from herbivory by arthropod pests [8].

MKs are a class of organic chemical compounds of natural origin [8] that can be successfully used as biopesticides. These compounds are commonly found on the leaves of tomato plants [9]. They account for up to 90% of the total secondary metabolites secreted by glandular trichomes [10]. Examples of MK components are as follows: 2-Dodecanone, a repellent component against *Rhipicephalus appendiculatus* (Neumann, 1901) and *Sitophilus zeamais* (Motschulsky, 1855) [11] found in the shrub *Cleome monophyla* (Linnaeus, 1753); 2-Tridecanone, responsible for the resistance of wild tomatoes (*Lycopersicon hirsutum* f. *glabratum* C. H. Mull., 1940) fighting multiple arthropods [12,13]; 2-Nonanone, found in the essential oils of the clove *Eugenia caryophyllata* (L. M. Perry, 1939) [14]; and 2-Heptanone, found in cinnamon oil and clove oil [15]. Plants and their products are not the only natural sources of this class of organic chemical compounds. MKs also occur as components of the odour secretions of insects, most commonly in the alerting pheromones of ants [8]. One example is 2-Heptanone, a volatile component which, at low concentrations, may act as a warning and recruiting pheromone, whereas at high concentrations, it functions as a repellent and alarm pheromone [8]. Although these properties could be exploited to develop biopesticides, until now, only 2-Undecanone is commercialised in the U.S. as an arthropod repellent [16] due to its intense repellent activity against mosquitoes and ticks [17]. Its insecticidal activity could also be used as a fumigant against arthropod pests, as demonstrated with the red imported fire ant (*Solenopsis invicta* Buren, 1972) [18]. It is important to highlight that most of the studies performed so far focus on insect pests by measuring classical parameters of toxicity, such as mortality or reproduction. However, works that focus on elucidating the toxicity and the mechanisms of action of defensive extracts are hitherto very scarce [19].

As with conventional insecticides, biopesticides should be tested for toxicity, selectivity, and unintended effects on human health and the environment before being introduced on the market. Pesticides can cause many physiological and biochemical changes when they enter the body, and deciphering the mode of action of pesticides can be one of the most reliable tools for predicting their effects [20]. Physiological and behavioural approaches may be combined with molecular methods to gain insight into mechanisms [21]. In addition, using multiple model organisms is also recommended for toxicity assessment approaches, in order to gain a deeper understanding of the toxic properties of a substance [22]. However, the studies on the effects of the secondary plant metabolites mainly focus on pest species; non-target organisms (pollinators such as bees and some natural enemies) have been comparatively less studied.

In this work, we tested the effect of 2-Dodecanone, a poorly studied methyl ketone, on *N. vitripennis* (Walker, 1836), a species highly suitable for testing and regarded as one of the most extensively studied model parasitoids [23]. Among Hymenoptera, parasitoid wasps are critical components as they provide vital ecosystem services to crops, such as pest control, playing an essential role in controlling arthropods of agricultural importance. The genome of the parasitoid wasp *N. vitripennis* has been sequenced [24] and has, during the course of the last decade, made significant contributions towards deepening our understanding of evolutionary biology, microbiome interaction, development, ecology, and behaviour. This species is sensitive to pesticides; for example, sublethal doses of the neonicotinoid imidacloprid disrupt sexual communication and host finding [25,26]. However, despite the ecological importance of parasitoids, there is little information on the toxicity of plant secondary metabolites and the risks to crucial insects from different taxa.

Given that molecular effects of MKs may have developmental and physiological consequences on a larger scale, which may ultimately affect the survival of the population, we investigated the toxic effects of 2-Dodecanone from the subcellular effect to the whole organism response in *N. vitripennis*. To determine whether this MK affects the wasps, we evaluate (i) the effect on gene expression related to endocrine pathways (ecdysone biosynthesis and ecdysone signalling pathways), mechanisms to cope with toxic stress (biotransformation processes and antioxidant responses), and cell homeostasis; (ii) the potential genotoxicity (comet assay); (iii) life history traits (time development and emergence rate); and (iv) the impact of its potential repellence on female reproduction and behaviour (egg-laying choice test).

## 2. Materials and Methods

### 2.1. Wasp Population

The *N. vitripennis* genome reference strain AsymC was used for this experiment [24]. They were maintained on pupae of *Calliphora* sp. at 25 °C under constant light and room humidity [27] since July 2015 in our laboratory [28].

### 2.2. Exposure Conditions, Short and Long-Term Assays

2-Dodecanone (CAS No: 6175-49-1; Sigma-Aldrich, St. Louis, MO, USA) was dissolved in acetone (CAS No: 67-64-1; purity 99.9%; Sigma-Aldrich) to obtain a stock solution of 1 g/L. Working solutions of 2-Dodecanone (5 and 500 μg/L) were freshly prepared for each experiment from the stock solution diluted in water containing 0.05% acetone. Females were exposed every day to a new solution. Experiments were carried out exclusively using females in the earliest phase of pupal instar (white pupae). These white pupae females were individually and topically exposed to the different conditions analysed in 48-well methacrylate plates by applying 0.5 µL of the solution to the dorsal thorax of the pupa, either during 48 h in the short-term exposure experiment (survival rate, gene expression, protein/carbohydrate/lipid content, ecdysteroids, genotoxicity test) or from 6 days until emergence in the long-term exposure experiment (development, emergence rate, and egg-laying test).

During short-term exposures, survival values were recorded at 24 h and 48 h. After 48 h, nine pools of 4 of 5 individuals (females in red pupae phase) per treatment (0.05% control acetone, 5 and 500 µg/L of 2-Dodecanone) were collected in 1.5 mL microtubes and frozen immediately on dry ice to subsequently measure gene expression, protein/carbohydrate/lipid content, and ecdysteroid levels. Fresh pupae exposed to control acetone or 500 µg/L 2-Dodecanone were also collected (3 per pool) to evaluate the genotoxicity of 2-Dodecanone. Five independent experiments were performed with 40 individuals per treatment each (*n* = 200 pupae/treatment).

Long-term exposures were performed with control acetone (0.05%) or with the highest concentration of 2-Dodecanone (500 μg/L). Every 24 h, the development phase of the pupal (white pupae, red-eye pupae, white and black pupae, and black pupae) and the adult stages were recorded. After adult emergence, the resulting females were used for the egg-laying test (see below). Six independent experiments were performed with 30 individuals per condition (*n* = 180 individuals/treatment).

### 2.3. RNA Extraction and cDNA Synthesis

For each sample analysed by qPCR (*n* = 3 for each treatment: 0.05% acetone, 500 µg/L 2-Dodecanone), total RNA was extracted from pools of 5 red pupae females using TRIzol Reagent (Invitrogen, Life Technologies, Carlsbad, CA, USA) following the manufacturer’s instructions. RNA was then treated with DNase I (Invitrogen, Life Technologies, Carlsbad, CA, USA) and extracted with phenol/chloroform/isoamyl alcohol (Fluka, Germany) using 5PRIME Phase Lock Gel Light tubes (Quantabio, Beverly, MA, USA). Purified RNA was resuspended in nuclease-free water, quantified by spectrophotometry at 260 nm using a BioPhotometer (Eppendorf, Hamburg, Germany), and stored at −80 °C. A total of 7 µg of RNA was reverse-transcribed in a 20 µL reaction system in a CFX96 Thermal Cycler (Bio-Rad, Hercules, CA, USA) using iScript™ Advanced cDNA Synthesis Kit (Bio-Rad), according to the manufacturer’s protocol.

### 2.4. Quantitative Real-Time PCR

Specific primer pairs for each gene were designed using Primer 3 (version 0.4.0) software [29] (Table 1).

The identities of the amplified fragments were confirmed by Sanger sequencing. Amplification efficiencies and correlation coefficients for each primer pair were calculated as described in the Real-Time PCR Applications Guide (Bio-Rad catalog #170–9799). For all genes, the efficiencies were between 90% and 105% (R^2^ > 0.980). Real-time quantitative PCR (qPCR) was performed using the QuantStudio 1 real-time PCR System (Applied Biosystems, Waltham, MA, USA) with the Power SYBR™ Green PCR Master Mix (Applied Biosystems), according to the manufacturer’s protocol. Genes encoding RPL6, RPL7, and Eef1 were used as endogenous references. Each qPCR was conducted in a 10 μL mixture containing 1 μL of sample cDNA, 0.6 μL of each primer (10 μM), 4.8 μL of nuclease-free water, and 5 μL of 2× Power SYBR™ Green PCR Master Mix. The qPCR cycling parameters were as follows: 95 °C for 5 min, followed by 40 cycles of 95 °C for 15 s and 60 °C for 30 s and 1 min elongation at 60 °C. Melting curve generation was performed from 60 to 95 °C. Data and Analysis software (Thermo Fisher Scientific, Waltham, MA, USA) was used for data extraction. The 2^−ΔΔCq^ Method [32] was used to determine total mRNA levels by normalising the expression of the target genes against the three reference genes. To check reproducibility, the qPCR for each sample was run in duplicate wells, and three technical replicates and three independent biological replicates were performed for each experimental condition.

### 2.5. Ecdysteroid Titration

Three biological replicates (pools of four red pupae for each treatment: 0.05% acetone, 500 µg/L 2-Dodecanone) were prepared for each experimental condition. After centrifugation at 9500× *g* for 10 min, the supernatant obtained was collected and dried under vacuum using a SpeedVac Concentrator (Eppendorf, Montesson, France). Samples were dissolved in a buffer for an Enzyme Immunoassay (EIA) adapted from Porcheron et al., 1989 [33]. The EIA titration was made using a polyclonal anti-20E antiserum L2 (dilution 1:62,500) and a 2-Succinyl-20-hydroxyecdysone coupled to peroxidase (dilution 1:100,000). O-Phenylenediamine, OPD, and hydrogen peroxide solution tablets (OPD, Sigma, St. Quentin Fallavier, France) were used for the chromogenic reaction, and optical density (OD) was measured at 450 nm. Calibration curves were produced for L2 antiserum using 20E (range from 16–2000 fmol; a gift from René Lafont, UPMC, Paris, France), and data are expressed as pg 20E equivalent/μL of hemolymph.

### 2.6. Energy Reserves

Three biological replicates (pool of 4 individuals) were frozen 48 h after each treatment (0.05% control acetone or 5 and 500 µg/L 2-Dodecanone) and then crushed in 230 μL of pure methanol before a 15 min centrifugation at 1500 rpm at 4 °C. A part of the supernatant (50 µL) was used to measure protein quantity. The remaining portion was mixed with methanol (final volume 350 μL) plus 150 μL of chloroform, left overnight at 4 °C, and then centrifuged for 15 min at 1500 rpm at 4 °C. A volume of 75 μL of supernatant was then used to quantify the concentrations of lipids and the same for carbohydrates. The protein content was measured using the Bradford assay (Sigma #B6916) from methanol extracts diluted into water. The absorbance of the sample was measured using a spectrophotometer at 595 nm, and the protein concentrations were determined from the standard curve of BSA (Bovine Serum Albumin, Sigma #B4287) (12.5–200 µg/mL) [34]. The total lipid content, consisting mainly of triacylglycerol, was determined in chloroform–methanol extracts by a vanillin–phosphoric acid reaction [35]. The lipid content was compared to a standard curve of sunflower oil diluted in chloroform (2–40 µg/mL). The absorbance of samples was measured spectrophotometrically at 525 nm. An anthrone procedure quantified the total carbohydrates in chloroform–methanol extracts [36]. The carbohydrate content was compared to a standard glucose curve diluted in ethanol 25% (2–40 µg/mL). The absorbance of samples was measured spectrophotometrically at 625 nm. The concentration of each element is expressed as a function of the individual’s weight.

### 2.7. Alkaline Comet Assay

Five pools of 3 individuals (red female pupae) per condition (500 µg/L 2-Dodecanone and 0.05% acetone) were gently mixed in PBS in ice to obtain cell suspension (*n* = 10). The conventional glass slides were dipped into Normal-Melting-Point Agarose (NMPA) (1%) (BioRad 161-3101) and air-dried. An amount of 20 μL of cell suspension was mixed with 70 μL of low-melting-point agarose (LMPA) (1%) (Sigma 3934681-1), spread on top of pre-coated slides, and maintained at 4 °C for 30 min. After solidification, slides were gently immersed in freshly prepared cold-lysing solution (2.5 M NaCl, 100 mM Na_2_EDTA, 10 mM Tris, pH 10) with 1% Triton X-100 and 10% DMSO for at least 1 h 30 min at 4 °C. One slide (positive control) per experiment was immersed in a solution of H_2_0_2_ 0.1 M for 15 min. After lysing, the slides were immersed in an electrophoresis buffer (300 mM NaOH and 1 mM EDTA) and left for 30 min to allow the unwinding of DNA and expression of alkali-labile sites. The slides were thereafter subjected to electrophoresis for 20 min at 300 mA and 1 Volt/cm. All steps were conducted at 4 °C and protected from light to prevent unintentional DNA damage. After electrophoresis, the slides were washed with PBS, and then H_2_O and ethanol for 5 min to neutralise. Slides were stained with 35 μL DAPI (1 μg/mL), covered, and observed at 400× in a fluorescent microscope (Olympus BX51, Tokyo, Japan) equipped with a 450–490 nm excitation filter. To calculate DNA damage, about 100 cells per sample were chosen randomly and analysed visually according to comet appearance. A total of 5 classes, i.e., from class 0 (no DNA damage) to class 4 (maximum DNA damage), give sufficient declaration [37]. Total comet score (TCS) was then calculated according to the formula TCS = 0(n) +1(n) +2(n) +3(n) +4(n), where “n” indicates the percentage of cells in each class.

### 2.8. Female Egg-Laying Test

We then measured whether exposure to 2-Dodecanone disrupted the oviposition behaviour of females and whether they could discriminate between contaminated and unexposed host patches. After emergence, each treated (500 µg/L 2-Dodecanone) and control (0.05% acetone) female from the long-term assays was put in the presence of a 1-day-old male for about 1.5 h for mating before being placed alone in a device for 24 h where they were allowed to oviposit in two host patches that were previously exposed to 2-Dodecanone or to 0.05% control acetone. In an experimental apparatus consisting of three plastic arenas (4 cm diameter) connected with small corridors (2.5 mm) both grounded with and covered by glass sheets, a patch of 5 host fly pupae exposed to 0.05% acetone was deposited in one of the external arenas, and a patch of 5 host fly pupae exposed to 500 µg/L 2-Dodecanone was deposited in the other external arena. Mated females exposed to 0.05% acetone or 500 µg/L 2-Dodecanone were set up in the middle arena (acetone: n = 51; 2-Dodecanone: *n* = 45) and left for 24 h in the apparatus before being removed. Fly host pupae were allowed to develop for 12 days before being opened to count the number of *N. vitripennis* pupae in each fly pupae.

### 2.9. Data Analysis

All analyses were performed using R Statistical Software (v4.1.2) [38]. We analysed the effect of the treatment on development using one Probit regression model (24 h) and a series of four linear regression models (GLM) fitted with binomial errors (48 h, 72 h, 96 h, and 120 h). In these models, we entered the developmental stage (0, 1, 2, 3, or 4) as the response variable, while we used the treatment (0.05% acetone, 5 µg/L and 500 µg/L 2-Dodecanone) as the explanatory factor.

Normality and homoscedasticity of model residuals were assessed with the Shapiro–Wilk and Levene tests, respectively. Normalised levels of transcripts were analysed with ANOVA, followed by Games Howell’s or Bonferroni’s post hoc tests, when appropriate. The Kruskal–Wallis test was used when data were not homogeneous or normally distributed, and the differences between pairs were established using Mann–Whitney tests. Probability (P) values of 0.1, 0.05, 0.01, and 0.001 were used as a cut-off for the statistical significance of differences between treatments and control samples. For the comet assay, the significance among the samples was compared at *p* < 0.05 with Kruskal–Wallis and Benjamini–Hochberg tests.

We conducted a generalised linear model (glm function in R) fitted with the quasibinomial error distribution, in which the response variable was whether the wasp laid in the patch or not (1 or 0, respectively), and the explanatory factor was the treatment (2-Dodecanone, acetone, or water). Because the treatment of females did not affect whether the wasps oviposited in the patch (see results), we tested whether females discriminated between treated and untreated hosts for oviposition using a binomial test on all females. The number of offspring per patch between treated and untreated females was finally compared using a Mann–Whitney rank test.

## 3. Results

### 3.1. Acute Toxicity of 2-Dodecanone

The survival rate was not affected by exposure to 2-Dodecanone at concentrations of 5 and 500 µg/L of 2-Dodecanone for 48 h (Mann–Whitney test; *p*-value = 0.6 and *p*-value = 0.8, respectively). Red pupae were collected after 48 h of exposure to both 2-Dodecanone concentrations to analyse gene expression, ecdysteroid quantity, energy reserves, and genotoxicity (acute toxicity studies). For the longest exposure (120 h) and the highest dose (500 µg/L 2-Dodecanone), no significant differences in survival rate were detected compared to the control condition (*p*-value = 0.638).

#### 3.1.1. Gene Expression Analysis

The transcriptional profile of different genes of interest under the selected experimental conditions was analysed through quantitative real-time PCR. For this purpose, *N. vitripennis* red pupae exposed to 0.05% acetone or 5 and 500 µg/L of 2-Dodecanone in acute (48 h exposure) toxicity studies were used.

Interesting dose-dependent alterations in the expression profiles were observed among the studied conditions. From a general perspective, most of the significant differences were observed in pupae exposed to the highest dose of 2-Dodecanone tested (500 µg/L).

##### Ecdysone Biosynthesis Pathway

**Figure 1:**
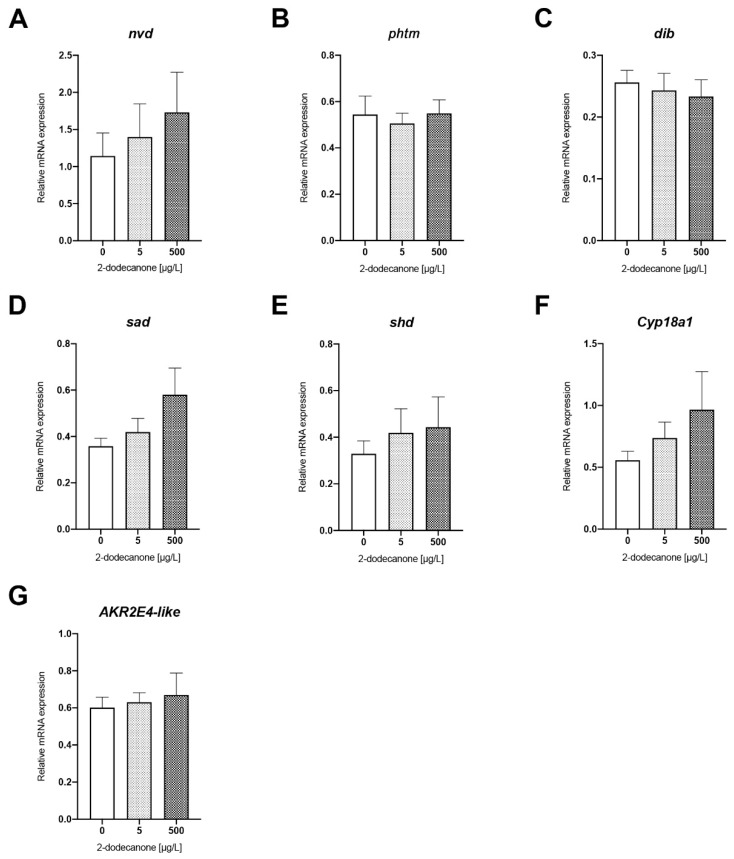
Changes in expression of genes related to ecdysone biosynthesis pathway in *N. vitripennis* red pupae exposed to 5 µg/L and 500 µg/L 2-Dodecanone for 48 h. Bars represent the expression patterns of the studied genes measured by real-time RT-PCR ± SE: (**A**) *nvd*; (**B**) *phtm*; (**C**) *dib*; (**D**) *sad*; (**E**) *shd*; (**F**) *Cyp18a1*; and (**G**) *akr2e4-like*. For each experimental condition, three independent experiments were performed, and RNA was extracted from groups of 5 pupae (*n* = 15). Significant differences with respect to control (Mann–Whitney test).

There were no significant alterations in the activity level of any of the genes involved in the ecdysone biosynthesis pathway (*nvd*, *phtm*, *dib*, *sad*, *shd*, *Cyp18a1,* and *akr2e4*) (ns; pairwise T Student Test, all *p* > 0.05) (Figure 1A–G). Finally, the level of ecdysteroids measured was not significantly different among conditions (Anova test, *p* = 0.603) between pupae that received 0.05% acetone and 5 µg/L or 500 µg/L of 2-Dodecanone for 48 h (Table 2).

##### Ecdysone-Signalling Pathway

**Figure 2:**
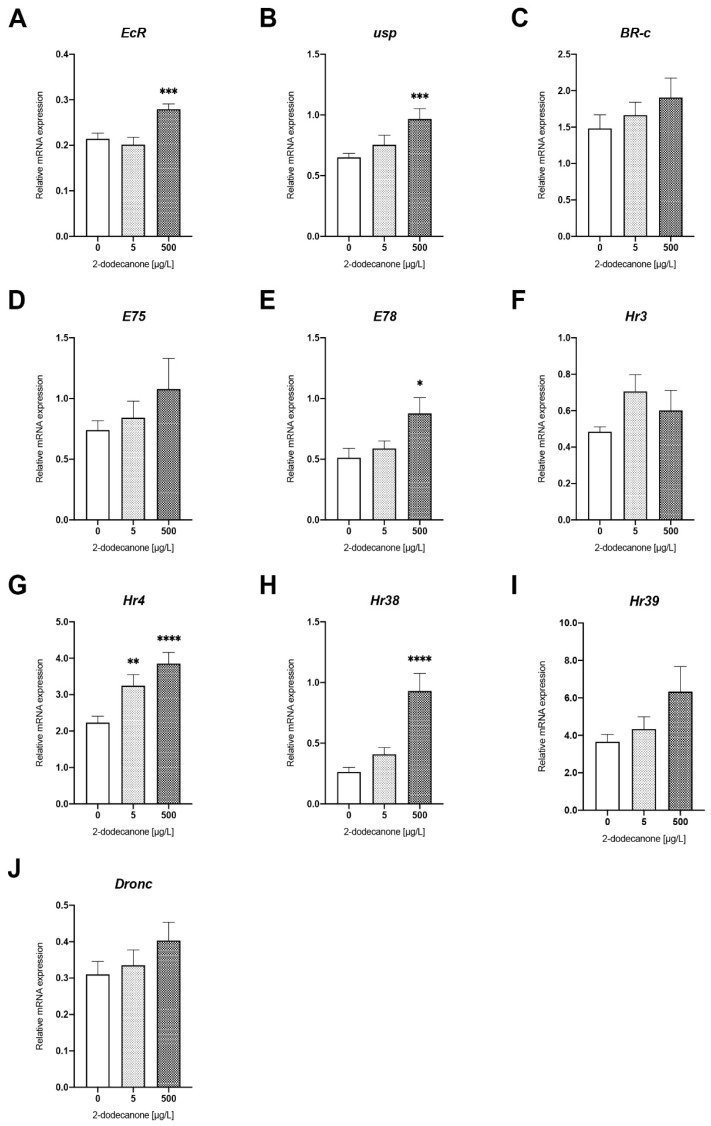
Changes in expression of genes related to ecdysone response pathway in *N. vitripennis* red pupae exposed to 5 µg/L and 500 µg/L 2-Dodecanone for 48 h. Bars represent the expression patterns of the studied genes measured by real-time RT-PCR ± SE: (**A**) *EcR*; (**B**) *usp*; (**C**) *Br-c*; (**D**) *E75*; (**E**) *E78*; (**F**) *Hr3*; (**G**) *Hr4*; (**H**) *Hr38;* (**I**) *Hr39*; and (**J**) *Dronc*. For each experimental condition, three independent experiments were performed, and RNA was extracted from groups of 5 pupae (*n* = 15). Significant differences with respect to control (Mann–Whitney test): * *p* < 0.1, ** *p* < 0.05, *** *p* < 0.01, and **** *p* < 0.001.

Most of the significant differences were observed in pupae exposed to the highest concentration of 2-Dodecanone used, compared to the control group. Genes coding for the heterodimeric ecdysone receptor (*EcR* and *usp*) (Figure 2A,B) were significantly upregulated in pupae exposed to 500 µg/L of 2-dodecanone, with a mean increase in transcription of 30% (*p* = 0.0073) and 48% (*p* = 0.0009), respectively, relative to the control condition. A concomitant response was also observed in some genes downstream of the ecdysone signalling pathway, with a significant upregulation of the early/late ecdysone response genes *E78*, *HR4,* and *HR38* after exposure to 500 µg/L of 2-Dodecanone (Figure 2E,G–I). The mean expression value of *E78* increased by 71% compared to non-exposed pupae (*p* = 0.0664). The gene coding for HR4 was upregulated at 5 µg/L and 500 µg/L, with a mean transcriptional increase of 45% (*p* = 0.0106) and 72% (*p* = 0.0006) relative to the control. *HR38* showed the highest induction, about 350% above the control, after exposure to 500 µg/L of 2-Dodecanone (*p* = 0.0002).

Finally, although not significant, a similar trend to upregulation was observed for *Br-C*, *E75*, *HR3*, *HR39,* and *dronc* genes, especially after 500 µg/L, with mean values up to 28%, 45%, 51%, 73%, and 30% above control values, respectively (Figure 2C–D,F,I,J).

##### Detoxification- and Homeostasis-Related Biomarkers

**Figure 3:**
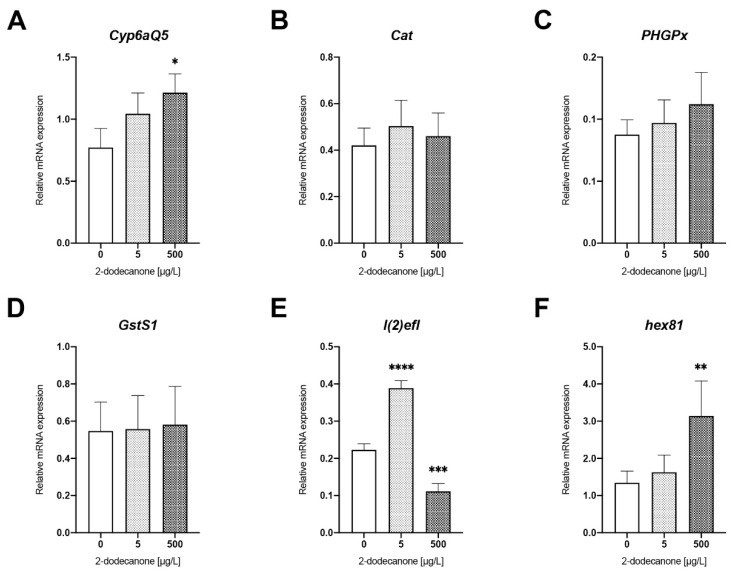
Changes in expression of genes related to biotransformation processes and homeostasis in *N. vitripennis* red pupae exposed to 5 µg/L and 500 µg/L 2-Dodecanone for 48 h. Bars represent the expression patterns of the studied genes measured by real-time RT-PCR ± SE: (**A**) *Cyp6aQ5*; (**B**) *Cat*; (**C**) *PHGPx*; (**D**) *GstS1*; (**E**) *l(2)efl*; and (**F**) *hex81*. For each experimental condition, three independent experiments were performed, and RNA was extracted from groups of 5 pupae (*n* = 15). Significant differences with respect to control (Mann–Whitney test): * *p* < 0.1, ** *p* < 0.05, ***p < 0.01, and **** *p* < 0.001.

2-Dodecanone affected the expression of genes involved in the antioxidant response differently. A time-dependent upregulation was observed for *Cyp6aQ5* with statistically significant changes at 48 h after exposure to 500 µg/L of 2-Dodecanone (*p* > 0.1; *p* = 0.0592) (Figure 3A). No differences were detected in the expression profiles of genes coding for enzymes related to the oxidative stress response: *Cat*, *PHGPx,* and *GstS1* (ns; *p* > 0.1) (Figure 3B–D).

Finally, two genes related to homeostasis processes were analysed: one coding the Protein lethal (2) essential for life (*l2efl*) and the other coding for a hexamerin (*hex81*). Our result showed a significant upregulation of *l2efl* with a mean value up to 51% above the control after exposure to 5 µg/L of 2-Dodecanone (*p* = 0.0003), followed by a strong repression of 68% compared to the control after exposure to 500 µg/L of 2-Dodecanone (*p* = 0.0041) (Figure 3E). Finally, a dose-dependent induction of *hex81* gene activity was also observed after exposure to 5 µg/L and 500 µg/L of 2-Dodecanone with a mean value up to 72% (ns *p* > 0.1) and 314% above the control (*p* = 0.040) (Figure 3F).

#### 3.1.2. Impact on Energy Reserves and Potential Genotoxicity

At the physiological level, the energy reserve in proteins, lipids, and carbohydrates of *Nasonia* pupae remained stable after 48 h of exposure, whatever dose of 2-Dodecanone was used (ns; pairwise *t* Student Test, all *p* > 0.05) (Table 2). The comet assay revealed no genotoxicity impact of 2-Dodecanone (500 µg/L) on red pupae after 48 h of exposition (Table 2), whereas the positive control H_2_O_2_ provoked significant total DNA damage (Kruskall–Wallis Chisq2 = 24.43, *p* = 6.54 × 10^−5^).

### 3.2. Long-Term Exposure to 2-Dodecanone

#### 3.2.1. Time Development and Emergence Rate

**Figure 4:**
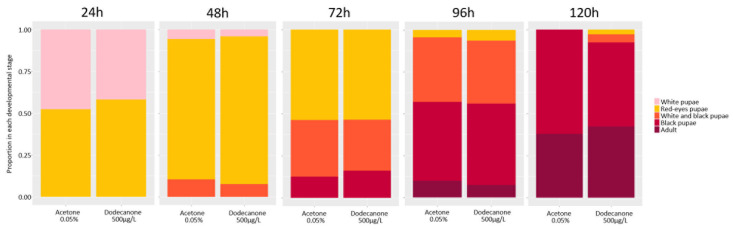
Effects of 2-Dodecanone on pupae development. The proportion of each developmental stage present over time up to 120 h after exposure of white pupae to 0.05% control acetone or 500 µg/L 2-Dodecanone. Each colour represents one developmental stage throughout the whole pupal stage (4 phases) and the adult stage. No significant differences (Anova test; *p* < 0.05).

As there was no difference in survival at 48 h (Table 2), the highest dose of 2-Dodecanone was selected to perform the following analysis on life traits, behaviour, and genotoxicity studies. The stage of development was analysed over 120 h (Figure 4), and whatever the treatment, the same proportion of each present stage was observed at each observed time (24 h: LRχ^2^_1_ = 0.636, *p* = 0.427; 48 h: LRχ^2^_1_ = 0.05, *p* = 0.825; 72 h: LRχ^2^_1_ = 0.02, *p* = 0.902; 96 h: LRχ^2^_1_ = 0.31, *p* = 0.580; 120 h: LRχ^2^_1_ = 2.10, *p* = 0.148). The emergence rate was calculated after long assays up to 144 h (6 days) of exposure, and no significant changes were observed after 500 µg/L of 2-Dodecanone compared to the control condition (Anova test *p* > 0.05; ns).

#### 3.2.2. Impact on Female Reproduction and Behaviour

**Figure 5:**
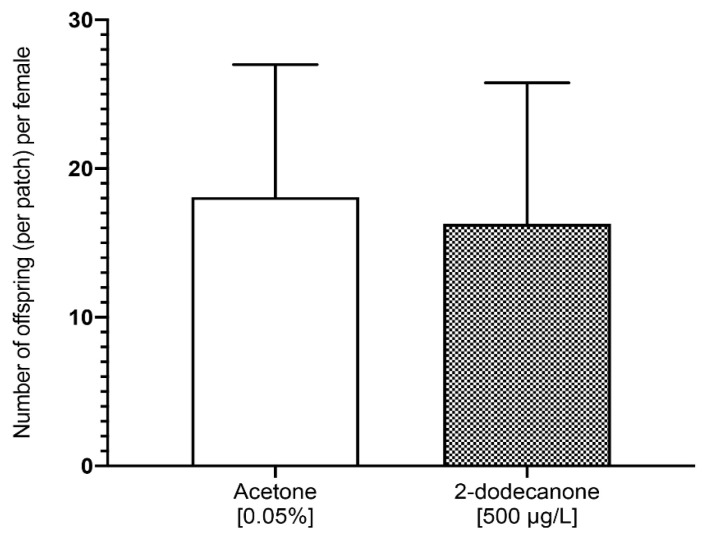
Effect of fly host exposure to 2-Dodecanone (500 µg/L) on the parasitism ability of *N. vitripennis* females regardless of the treatment they received during their development. The number of pupae of *N. vitripennis* found in a patch of fly host pupae treated with 0.05% acetone (control) or 500 µg/L 2-Dodecanone after adult females of *N. vitripennis* were given a choice to lay eggs for 24 h in these fly host pupae, treated or not. Bars represent mean ± SE. No significant differences (Mann–Whitney rank test; *p* < 0.05).

We tested the long-term effect of 2-Dodecanone on the egg-laying host selection behaviour and reproduction of exposed females during their pupal development. The preference of *N. vitripennis* adult females for either treated or non-treated host fly pupae was evaluated through host-choice bioassays under free-choice conditions. Our results showed that regardless of the female’s treatment during their pupal stage, they chose a patch of host and laid their eggs in a single patch (only 1 out of 84 females laid eggs in two patches). Treatment of females did not affect their choice to lay eggs in a patch of fly host treated or not with 2-Dodecanone (GLM; Likelihood Ratio Chisq2 (2) = 0.25; *p* = 0.882). Whatever the treatment they previously received, females did not prefer to lay eggs in control fly pupae or those treated with 2-Dodecanone (binomial test; *p* = 0.11). They laid the same number of offspring in the host patches in both conditions (Figure 5) (Mann–Whitney; W= 847.5; *p* = 0.259).

## 4. Discussion

Biopesticides (natural substances purified from living organisms) are viewed as an environmentally friendly alternative to the use of conventional (synthetic) pesticides to control pests [39]. Plants are the most efficient source of natural pesticides; it synthesises numerous products, many of which have been shown to affect insects and other harmful organisms. Some are highly toxic to a wide range of organisms, including both vertebrates and invertebrates. However, most secondary metabolites affect insects and are comparatively harmless to other taxa. Despite their potential as biopesticides, secondary metabolites on the insect’s biochemical mode of action are still obscure at the molecular level. To better understand their spectrum of action and biocidal potential, with implications at the agronomic and environmental levels, it is necessary to elucidate their mechanisms.

Our experimental results showed for the first time in insects that the plant allelochemical 2-Dodecanone alters expression profiles of different genes involved in the ecdysone response pathway, biotransformation metabolism, and cell homeostasis.

This work revealed that 2-Dodecanone triggers the ecdysone response pathway at the transcriptional level. This is a major result as disruption of the ecdysone pathway regulates cellular changes during moulting and metamorphosis [40,41]. In the hymenopteran parasitoid we studied, 2-Dodecanone behaved as an ecdysone mimetic at acute/short term exposures (48 h). Exposure to 500 µg/L of 2-Dodecanone led to a significant induction of both *EcR*—a well-known biomarker of endocrine disruption—and *usp* heterodimer partners. Overexpression of the nuclear ecdysone receptor gene was concomitant to the induction of most genes downstream of the ecdysone signalling pathway, significantly affecting *E78*, *Hr4*, and *Hr38*. The effects observed were not related to changes in the levels of ecdysteroids, which remained constant after exposure to this MK. In addition, the expression of genes involved in the synthesis of ecdysteroids was not affected. Furthermore, while the *EcR* promoter contains an ecdysone response element that can increase its transcription in the presence of ecdysteroid, the same cannot be stated for *usp*. During normal development, *usp* mRNA levels remain essentially unchanged [42,43]. The ability of 2-Dodecanone to induce *usp* expression was unexpected, and it contrasts with most other endocrine-disrupting compounds (EDCs) that have been tested in our laboratory with the model dipteran *Chironomus riparius* (Meigen, 1804) [44,45,46,47,48,49,50]. To the best of our knowledge, there are no previous data about the molecular effects of 2-Dodecanone on the endocrine system of insects. Our results also showed that 2-Dodecanone acts differently to 2-Tridecanone, for which a decrease in the expression of the gene related to 20-hydroxyecdysone biosynthesis has been reported in *Helicoverpa armigera* (Hübner, 1808) [19]. In this sense, further studies are needed to elucidate whether MKs may exert different toxicity depending on their size and specific properties.

To protect against the effects of toxicants and oxidative stress, organisms have a variety of detoxifying enzymes, such as catalase (CAT), glutathione-s-transferase (GST), glutathione peroxidase (GPx), or cytochrome P450 monooxygenases (P450s) [51]. The early detection of variations in the pattern of genetic activity is a very useful approach to analyse the effects of exposure to stress factors [46,52,53,54]. However, to date, very limited information is available about molecular biomarkers in non-pest insects exposed to pesticides or in phytophagous insects exposed to the secondary metabolites of their host plants [19,55]. Detoxification systems are one of the different physiological pathways that may be affected by toxicants. For example, recent studies have described the oxidative stress induced by pesticides in honeybees [56,57]. In this sense, antioxidant enzymes such as superoxide dismutase (SOD) and catalase (CAT) are crucial in defending organisms against such stress [58] and have been associated with the toxicity of pesticides in insects [59]. Cytochrome P450 monooxygenases (P450s, and CYP3 and CYP4 clades) constitute essential metabolic systems since they are involved in the oxidative detoxification of plant secondary metabolites and synthetic insecticides. In our experiment in *N. vitripennis*, no significant differences were detected in pupae in antioxidant genes, such as *GstS1*, *Cat,* and *PHGPx*, but an induction of cytochrome p450aQ5 was observed after acute exposures to 2-Dodecanone. Our data suggest that the compensation mechanism against 2-Dodecanone intoxication might imply CYP genes of Phase I in biotransformation processes more than genes related to antioxidation defence [60]. Future studies are planned to deepen the effect of 2-Dodecanone in the biotransformation response through the analysis of other p450 cytochromes in *N. vitripennis*. It is worth noting that the number of glutathione S-transferase, cytochrome P450, and esterase genes in the genome of the hymenopteran parasitoid *N. vitripennis* is about twice that found in the genome of another hymenopteran (the honeybee *Apis mellifera* (Linnaeus, 1761)) [61], probably because they are thought to encounter a wider range of potentially toxic xenobiotics in their diet and habitat [62]. Thus, activating such a metabolic detoxification pathway could explain the low phenotype observed in this species. For other insect species, such as social species less well equipped with detoxification systems, the consequences could be more critical regarding survival, development, or other altered physiological functions.

2-Dodecanone did not affect larval survival, development, or energy reserves in *N. vitripennis*, even though some molecular biomarkers were modulated. At this point, further studies are needed to elucidate whether some detrimental changes at this level might occur over the long term. Nevertheless, compensatory systems at the molecular level may be put in place to minimise the phenotypic consequences for exposed individuals. Interestingly, the overexpression of two poorly known genes, *hexamerin* and *lethal (2) essential for life*, in response to 2-Dodecanone exposure could reflect this compensation system. Hexamerin proteins (Hex) have been described to function mainly as storage proteins that supply amino acids for insect metamorphosis [63]. The existence of a variety of genes encoding hexamerins in several insect species supports the idea that they evolved distinct roles due to physiological adaptations [63]. Our results showed a dose-dependent and up-regulation of *hex81*, up to 314% compared to the control after 48 h of exposure to 500 µg/L of 2-Dodecanone. The atypical pattern of transcript accumulation during the pupal stage could suggest that the hexamerin encoded by *hex81* has an additional role in the *N. vitripennis* life cycle, mediating gonad differentiation and gamete formation and, by extension, reproduction. Egg production in *N. vitripennis* females may occur at the expense of hexamerins (and other compounds) stored during the larval feeding stage [64]. It is also known that some ant species use larval hexamerins as amino acid sources to produce the first batch of worker-destined eggs [65]. Considering that *N. vitripennis* females lay eggs soon after emergence, they may use larval hexamerins for this purpose. It is worth highlighting that no changes were detected in protein reserves by 2-Dodecanone, but a strong *hex81* induction took place after the acute toxicity test. These results suggest that a new pool of transcripts could be synthesised to compensate for other potentially detrimental effects on growth and reproduction led by 2-Dodecanone. Finally, the gene *lethal (2) essential for-life (l(2)efl)* encodes a member of the heat shock 20 protein (HSP20) family. HSPs are chaperones induced during stress (including heat shock, pathogen infection, heavy metal ion exposure, hypoxia, and osmotic stress), and they act as chaperones to guide misfolded proteins [66]. Our findings showed that 2-Dodecanone led to different significant changes in the expression pattern of *l(2)efl* depending on the doses, with an up-regulation after 5 µg/L of exposure, followed by a strong repression of 65%. This gene did not show differential accumulation in mosquitoes exposed to insecticides or pollutants [67]. Insect small HSPs also play essential roles in the development regulation, as described in the cigarette beetle, *Lasioderma serricorne* (Fabricius, 1792), where LsHsp19.4 and 20.3 may have crucial functions in pupal formation [68]. Similar roles of small HSPs have been observed in *Drosophila melanogaster* (Meigen, 1830) [69], *Plutella xylostella* (Linnaeus, 1758) [70], *Liriomyza sativae* (Blanchard, 1938) [71], and *Spodoptera litura* (Fabricius, 1775) [72]. In *Apis cerana* (Fabricius, 1793), the *Hsp22* gene was significantly upregulated by abiotic and biotic stresses, such as xenobiotics, cyhalothrin, pyridaben, UV, CdCl_2_, 20-hydroxyecdysone, and Ascosphaera apis [(Maaßen ex Claussen) L.S.Olive & Spiltoir, 1955] treatments [73]. Taking together the essential roles of *l(2)efl* in development, immunity, and stress, the repression observed in our work might affect the developmental progression of *N. vitripennis*.

Regarding the mechanisms of action of pesticides, their possible genotoxic effects have mainly been focused on mammals for the purpose of analysing the effects they may have on consumers and workers that employ them; however, their effects on insects are much understudied [74,75]. The single-cell gel electrophoresis assay (SCGE), also known as the comet assay, has gained widespread recognition due to its high sensitivity in detecting very low levels of DNA damage (DNA strand breaks, alkali-labile sites, and strand breaks associated with incomplete excision repair sites) in single cells, in research studies or for regulatory purposes [76,77,78,79]. The comet assay presents several advantages over other commonly used assays for genotoxicity studies [80]. Also, in this study, we analysed using the Comet assay whether 2-Dodecanone altered the DNA of wasps exposed during larval life, and no effect was observed, which is reassuring regarding the potential use of 2-Dodecanone as a biopesticide.

Parasitoids are natural enemies of insect pests, and ecologically, their destruction could unbalance agroecosystems and have consequences on the population dynamics of the pests they control. Therefore, it is important to analyse the potential effects of pesticides on the parasitoids’ own characteristics, i.e., their ability to parasitise hosts. Indeed, among the sublethal effects, some pesticides can alter the parasitism rate, as shown for pyrethroids on *Trichogramma pretiosum* (Riley, 1879) [81] or fenitrothion and deltamethrin on *Trissolcus grandis* (Thompson, 1861) [82]. Our results show that 2-Dodecanone does not appear to affect the ability of females detecting a host, since the treated females parasitise as many hosts as untreated females. Similarly, the rate of parasitism is not affected by 2-Dodecanone, since females laid an equal number of eggs in each host fly pupa. Contrary to other MKs as 2-Undecanone [16], 2-Dodecanone does not appear to be a repellent for females, since females lay the same number of offspring in fly pupae hosts exposed to 2-Dodecanone or the solvent control.

Our work reveals the interest in utilising 2-Dodecanone as a natural defence for plants against phytophagous insects, and the need to further investigate its mechanisms of toxic action. The next step in our study will be to analyse the effects on pest insects to determine whether they are more harmful than in non-target species, as well as to deepen the mechanism of action in other model insects (terrestrial and aquatic) commonly used in our laboratories.

## 5. Conclusions

For the first time, this study provides evidence that 2-Dodecanone can alter the transcriptional activity of genes related to ecdysone-related pathways, the biotransformation process, and cell homeostasis in insects. Our understanding of how 2-Dodecanone works has therefore improved. Moreover, despite the activation of the ecdysteroid response pathway, there were no phenotypic effects on development or the physiology of behaviour in a parasitic hymenoptera. This is reassuring regarding the absence of unintended effects that 2-Dodecanone might induce. In addition, our research highlights the potential suitability of *N. vitripennis* for the in-depth study of the molecular effects of plant defence mechanisms in insects. The combination of molecular tools and multi-organism testing, with both non-target and pest species, could lead to more robust toxicological evaluations and environmental risk assessments as well as a better understanding of the biocidal potential that MKs may bring to pest management.

## Figures and Tables

**Table 1:** Primers used for cDNA sequencing and real-time qPCR of genes studied in *N. vitripennis* (*Nv*). Forward (F) and reverse (R) sequences, length of amplified fragments, and origin of primers.

	Gene	Primer Sequence (5′-3′)	Gene Reference
**ECDYSONE BIOSYNTHESIS** **PATHWAY**	***nvd***	**F** GCCTGGATACGAGATTTAACG **R** CCAACCATTCGGGTAAACTG	NASONIABASE: NV17010
	***phtm***	**F** TCAAGTTTCCCAGTCCCAAG **R** ACGATGAAGACGACGAGCTT	XM_032601767.1 NASONIABASE: NV19009
	***dib***	**F** CTTTATGCCGGAGCGATG **R** GCGACACATCCTGAGCAGT	XM_001601625.6 NASONIABASE: NV14828
	***sad***	**F** GCCGGTGACACTACAGCTTAC **R** TAGAGCCGTAGCGACTCCTT	XM_008214583 NASONIABASE: NV11849
	***akr2e4***	**F** GCCGAAGGTAGACGAAATCA **R** GGCCAATTTGACAGCATTTT	XM_001603888.5 NASONIABASE: NV13784
	***shd***	**F** GCCTCGAGAGATGCCTTCTA **R** AACTGCTTCGATGCTCTCGT	NM_001172547.1 NASONIABASE: NV17940
	***Cyp18a1***	**F** ACGACTCGCGAGGTCAATCT **R** GAAGTACTCGGGCTTGTGGA	XM_001600748.5 NASONIABASE: NV19010
**ECDYSONE SIGNALING** **PATHWAY**	***EcR***	**F** GCCCAGAAGGAGAAGGACAAA **R** CGTTGAGCGAGCCTATGGAG	NM_001159357.1 NASONIABASE: NV11088
	***usp***	**F** GTCCTGGGTCTTTAAACCTTG **R** CAGAGATGTTTGCTGCCAGA	XM_001605769.6 NASONIABASE: NV15186
	***E75***	**F** CGACACTGGCAACCTGACTG **R** GCACCGCATCACGACTCAT	NASONIABASE: NV50220
	***BR-C***	**F** AGCTTCTCAAGAGCA **R** CTACTGAGAGAGCGCTGGTG	XM_008211399.2 NASONIABASE: NV15002
	***E78***	**F** CTCGACTCAGAACGCGATGA **R** CCTGGTGGACTCGTCGGTAA	XM_016987036.1 NASONIABASE: NV13864
	***Hr3***	**F** AAGCAGGAGACGACGACCAC **R** CGCAAGGGAGGACATACTGG	XM_016986225.1 NASONIABASE: NV50123
	***Hr4***	**F** GGAGGGTGAAACTGAGGATGG **R** ACTTCGGGCAATCTGACGAG	XM_008206002.2 NASONIABASE: NV14578
	***Hr38***	**F** GACGATACGCAGGGAGGAGA **R** CGAGCTTGCACATGGAGATG	HR3. XM_001601528.4 NASONIABASE: NV14578
	***Hr39***	**F** GATCTCAAGTCCCATCGCCA **R** TCGAGGCGTCCGAGTTATTG	XM_001603476.5 NASONIABASE: NV13389
	***Dronc***	**F** GAAACTGAAATCCAAGGCATCG **R** GGCAAATCGTGAAACAACAGC	XM_032598125.1 NASONIABASE: NV15018
**BIOTRANSFORMATION PROCESSES AND HOMEOSTASIS**	***Cyp6aQ5***	**F** GGAAATCGACGAAAAAGTTGG **R** TTTGCAGGTAACGCATGAAA	NM_001172525.1 NASONIABASE: NV13220
	***Cat***	**F** CGTGATCTTCGTGGTTTTGCTG **R** GGATTGGATCGCGGATGAAG	[30]
	***PHGPx***	**F** AAGTGTGGTTACACAGCTAAGCATT **R** GATATCCAAATTGATTACACGGAAA	[31]
	***GstS1***	**F** GTGTGACCAGCAACGAATGG **R** TCCAGATCCTCCCATGTGCT	From *Prodiamesa olivacea* (Diptera; data unpublished)
	***Ie2ef1***	**F** TCGAAGGAAAGCACG **R** GTGATGCTGAGGACT	XM_032597114.1 NASONIABASE: Nv13797
	***hex81***	**F** CAACAAGGAAGCAGT **R** GTGTCGAAGTCCTTG	NASONIABASE: NV12472
**REFERENCE GENES**	***RpL6***	**F** AAGAAGACACCCAAGAAGGAA **R** ACAATGGGATCTGAGGTAGGA	NM_001159919.1 NASONIABASE: NV12167
	***RpL7***	**F** AAGAAAGTCGAGCCCAAGAAG **R** GGCTGAATATCCTCGGCAAT	NASONIABASE: NV14954
	***EF-1a***	**F** CACTTGATCTACAAATGCGGTG **R** CCTTCAGTTTGTCCAAGACC	NM_001172756.1 NASONIABASE: NV13182

**Table 2:** Effect of exposure to 2-Dodecanone (5 or 500 µg/L) or 0.05% acetone on the pupal survival of *N. vitripennis*; ecdysteroid concentration; protein, lipid, and carbohydrate contents; and genotoxicity (comet assay) after 48 h exposition and on emergence rate after 120 h exposition. The number of independent experiments (exp) and the number of individuals (ind) are specified.

	Control Acetone (0.05%)	2-Dodecanone 5 µg/L	2-Dodecanone 500 µg/L
Pupal survival rate (%) (48 h)	97.9 ± 2.9 (4 exp, 114 ind)	96.9± 2.9 (4 exp, 107 ind)	95.3 ± 0.7 (4 exp, 114 ind)
Ecdysteroids (fmol/mg)	550 ± 124 (3 exp, 12 ind)	653 ± 92 (3 exp, 12 ind)	704 ± 177 (3 exp, 12 ind)
Proteins (µg/mg/individual)	6.2 ± 0.7 (3 exp, 12 ind)	6.1 ± 0.8 (3 exp, 12 ind)	6.3 ± 0.7 (3 exp, 12 ind)
Lipids (µg/mg individual)	13.7 ± 3.8 (3 exp, 12 ind)	11.4 ± 2.3 (3 exp, 12 ind)	14.0 ± 4.8 (3 exp, 12 ind)
Carbohydrates (µg/mg/individual)	2.2 ± 1.3 (3 exp, 12 ind)	2.2 ± 0.4 (3 exp, 12 ind)	2.1 ± 0.9 (3 exp, 12 ind)
Comet assay	155 ± 57 (10 exp, 30 ind)	-	133 ± 56 (10 exp, 30 ind)
Emergence rate (%) (120 h)	57.1% ± 1.8 (5 exp, 105 ind)	-	49.2% ± 2.1 (5 exp, 120 ind)

## Data Availability

The original data presented in the study are included in the article; further inquiries can be directed to the corresponding authors.
